# Advances in the diagnosis of acute pulmonary embolism

**DOI:** 10.12688/f1000research.21347.1

**Published:** 2020-01-24

**Authors:** Ella Ishaaya, Victor F. Tapson

**Affiliations:** 1 Department of Medicine, Drexel University College of Medicine, Philadelphia, PA, 19129, USA; 2 Division of Pulmonary and Critical Care Medicine, Cedars-Sinai Medical Center, Los Angeles, CA, 90048, USA

**Keywords:** pulmonary embolism, thrombosis, computed tomographic angiography, diagnosis, symptoms, risk factors

## Abstract

Venous thromboembolism is a common disease which remains underdiagnosed because of nonspecific presentations which can range from asymptomatic incidental imaging findings to sudden death. Symptoms can overlap with comorbid cardiopulmonary disease, and risk factors that offer clues to the clinician are not always present. The diagnostic approach can vary depending on the specific clinical presentation, but ruling in the diagnosis nearly always depends on lung imaging. Overuse of diagnostic testing is another recognized problem; a cautious, evidence-based approach is required, although physician gestalt must be acknowledged. The following review offers an approach to the diagnosis of acute pulmonary embolism based on the assessment of symptoms, signs, risk factors, laboratory findings, and imaging studies.

## Introduction

Venous thromboembolism (VTE), which includes the spectrum of deep venous thrombosis (DVT) and pulmonary embolism (PE), is the third most common cardiovascular diagnosis following myocardial infarction and stroke. Acute PE causes approximately 100,000 deaths per year in the United States ^1^. Unfortunately, the diagnosis is complicated by nonspecific clinical presentations, which can range from incidental imaging findings to sudden death. As such, timely diagnosis is critical but challenging, and PE remains one of the most commonly underdiagnosed conditions ^2, 3^. Although diagnosis rates have improved, PE is commonly not diagnosed or even suspected until after the patient dies ^4, 5^. While the diagnostic evaluation is intimately associated with risk stratification, which may impact on the level of therapeutic aggressiveness, we will focus on the diagnostic approach to acute PE.

## Pathophysiology of acute pulmonary embolism

The vast majority (95%) of acute PE cases originate from thrombi in the leg or pelvic veins, although emboli may arise from other sources such as the axillary subclavian system or the renal veins ^6^. These thrombi dislodge and embolize to the pulmonary arteries, causing obstruction to the pulmonary capillary bed and subsequent hemodynamic abnormalities. They also promote the release of vasoconstrictors, which increase pulmonary vascular resistance and right ventricular (RV) afterload. As the embolic burden increases, RV afterload increases and there is RV dilation and hypokinesis. When the clot burden reaches a critical threshold, the RV is unable to generate enough force to achieve an adequate cardiac output and fails, resulting in hypotension and cardiac arrest. RV pressure overload may also lead to ischemia due to compromised left ventricular filling, increased wall stress, and limited myocardial oxygen supply ^7^. Furthermore, significant pulmonary vascular obstruction leads to increased dead space and hypoxemia ^7^. These events translate into clinical findings that can offer clues to the diagnosis.

## Risk factors

Confirming the diagnosis of acute PE or refuting it often depends on lung imaging but relies on the history or clinical suspicion, physical exam, lab testing, and sometimes scoring systems. Risk factors can offer helpful clues, but their absence does not rule out the diagnosis. Acute VTE is clearly provoked but very often unprovoked or in a gray zone in between. Some risk factors are more influential than others. Most cases of acute VTE develop because of a combination of risk factors arising from Virchow’s triad of stasis, venous injury, or hypercoagulability (thrombophilia) ^6– 9^. Inherited and acquired thrombophilias increase the relative VTE risk by two to threefold, and such patients are often younger and may have a history of recurrent spontaneous miscarriages and/or a family history of VTE ^7, 10^. Acquired risk factors are more prevalent and may be helpful in leading to a diagnostic evaluation for acute VTE ( Table 1).

**Table 1.** Risk factors for venous thromboembolism (VTE)*.

**Hereditary factors ^†^**
Antithrombin deficiency
Protein C deficiency
Protein S deficiency
Factor V Leiden
Activated protein C resistance without factor V Leiden
Prothrombin gene mutation
Plasminogen deficiency
Dysfibrinogenemia
Acquired factors *
Reduced mobility
Advanced age
Cancer
Acute medical illness
Major surgery
Trauma
Spinal cord injury
Pregnancy and the postpartum period
Oral contraceptives
Hormone replacement therapy
Polycythemia vera
Antiphospholipid antibody syndrome
Heparins (heparin-induced thrombocytopenia)
Chemotherapy
Obesity
Central venous catheterization
Immobilizer or cast
**Probable factors**
Elevated homocysteine
Elevated factors VIII, IX, and XI
Elevated fibrinogen
Elevated thrombin-activated fibrinolysis inhibitor
Low levels of tissue factor pathway inhibitor

*In a compatible clinical setting, acute deep vein thrombosis and/or pulmonary embolism should be considered even in the absence of known risk factors. ^†^It remains unclear whether some of the disorders listed above are hereditary, acquired, or both.

Recent major surgery (defined as surgery that required endotracheal intubation or epidural anesthesia) is associated with an up to fivefold increase in VTE risk owing to prolonged immobilization and activation of pro-inflammatory substances and the coagulation cascade ^11^. A large prospective study on middle-aged women found a greater than 100-fold increase in VTE incidence in the first six weeks following surgery ^12^.

Immobilization, which includes prolonged travel, increases risk for VTE due to venous stasis. A retrospective study on critically ill patients found that central venous catheter use, mechanical ventilation, and reduced mobility increased in-hospital VTE occurrence by 1.7% in patients on anticoagulant prophylaxis ^13^. Changes in our society appear to be affecting the risk of acute VTE. For example, over the past two decades, there has been a documented increase in pediatric VTE, potentially attributable to prolonged immobilization, i.e. video game use ^14^.

Previous VTE increases the risk of recurrence two to threefold and often occurs within the first six months off anticoagulation. Oral contraceptives increase the risk for VTE ^15^. Oral contraceptive users, especially third and fourth generation, are at greater risk for VTE compared to non-users ^15^.

Active cancer increases the risk of developing VTE by five to sevenfold and is associated with a worse prognosis ^16^. Tissue factor, a tumor-derived protein, initiates the extrinsic pathway of the coagulation cascade and has been associated with increased VTE in pancreatic and ovarian cancer due to direct induction of a hypercoagulable state ^16, 17^. Additionally, tumor cells secrete pro-inflammatory cytokines and growth factors like tumor necrosis factor alpha and vascular endothelial growth factor, which are known to promote coagulation ^18^. Cancer patients also typically undergo surgery and chemotherapy and experience prolonged periods of immobilization, all of which increase their risk for VTE development.

Additional cardiopulmonary conditions, like chronic obstructive pulmonary disease and congestive heart failure, are associated with increased risk for VTE and may complicate the diagnostic picture ^19– 21^. PE can be mistaken for exacerbations of these disorders because of similar clinical presentations (dyspnea, chest pain, and elevated neck veins) ^22^. An emergency department study found that PE diagnosis was delayed in one-third of 436 cases; the delay was significantly more common in patients with chronic obstructive pulmonary disease (29.7% versus 7.25%) ^3^. In summary, underlying risk factors should lead to consideration of acute PE, in compatible clinical circumstances, and caution should be undertaken to be certain that PE is not overlooked simply because of a concomitant cardiopulmonary comorbidity.

## Symptoms and signs

Common symptoms of acute PE, including dyspnea and chest pain, are nonspecific and occur in many other cardiopulmonary diseases ( Table 2).

**Table 2.** Symptoms and signs in patients presenting with acute pulmonary embolism (PE) *.

**Common symptoms (>50%)**
Dyspnea
Sudden-onset dyspnea
Pleuritic chest pain
**Less-common symptoms (16–49%)**
Cough †
Lightheadedness/presyncope
Syncope
Leg swelling/pain
**Rare symptoms (<15%)**
Gradual onset of dyspnea
Orthopnea
Hemoptysis
Angina-like chest pain
Palpitations
Wheezing
**Signs**
Visible anxiety
Fever
Tachycardia
Tachypnea
Hypotension
Chest wall tenderness
Leg swelling/tenderness
Wheezing
Signs of overt right ventricular failure (e.g. neck vein distension, right ventricular S3)

*Symptoms vary based upon the embolic burden and physiologic response to the embolism as well as upon the presence or absence of underlying cardiopulmonary disease. ^†^Both cough and fever in the setting of acute PE are often due to non-PE related comorbidities. This table was created based upon data from 21, 25.

Sudden-onset dyspnea is the most common presentation ^21^. In the setting of pulmonary infarction, pleuritic chest pain is common, and rarely hemoptysis may occur. Cough may be present but is often related to other underlying conditions. PE may be asymptomatic, present atypically, or develop gradually over the course of weeks ^23^. Of note, roughly one-third of patients presenting with DVT have concomitant PE, even when asymptomatic ^24^. With high-risk (massive) and intermediate-risk (submassive) acute PE affecting RV function, lightheadedness and/or syncope may occur. A study of elderly patients found that this cohort more frequently presented with syncope than more classic symptoms like chest pain (33% versus 7%) ^26^. VTE incidence increases exponentially with age, thus increasing the importance of recognizing atypical PE presentations. A study examining differences between males and females with PE found that women are more likely to present atypically with symptoms like syncope ^27^. Acute PE is often overlooked in critically ill patients, since common symptoms are often overlooked or blamed on other underlying conditions. Signs of acute PE include nonspecific features such as visible anxiety, tachycardia, tachypnea, or hypotension. Contrary to popular teaching, chest wall tenderness can occur in acute PE because of pulmonary infarction ^28^. Clinicians should consider other evidence, such as increased oxygen demand, for the diagnosis of PE in this cohort ^29^.

## Diagnostic approach

### Clinical probability models

The combination of symptoms and clinical findings with the presence of predisposing factors for VTE allows the classification of patients with suspected PE into different categories of pre-test probability, which correspond to an increasing prevalence of confirmed PE. This pre-test assessment can be done either by clinical judgment/gestalt or via the use of prediction rules. Several prediction models exist for the evaluation and risk stratification of PE. The most widely studied include the Wells score ^30^ and the series of Geneva scores ^31– 33^, both of which aim to minimize invasive diagnostic testing. The simplified Geneva score, attributing one point per variable (except heart rate >95 beats/minute = 2 points) was prospectively validated in outpatients in the ADJUST-PE management outcome study, facilitating its effective use in the management of suspected PE ^33^.

The Pulmonary Embolism Rule-out Criteria (PERC) rule was developed to exclude PE and to minimize unnecessary diagnostic testing ^34, 35^. When a patient is deemed to have low gestalt clinical suspicion for PE and the eight criteria are absent (age <50 years, pulse <100 beats/minute, SaO _2_ >94%, no unilateral leg swelling, no hemoptysis, no recent trauma or surgery, no history of VTE, no estrogen use), it is suggested that the diagnosis of PE should not be pursued ^34, 35^. Inclusion criteria for a prospective validation of the PERC rule in the United States were new-onset or worsening of shortness of breath or chest pain and a low clinical probability of PE. It was found that of the 24% of patients excluded by the PERC rule, 1.3% were found to have PE ^35^. Naturally, “low gestalt clinical probability” is subjective. The PERC rule was validated in the European PROPER trial ^35^; however, the low overall prevalence of PE in these studies warrants caution regarding the generalizability of the results and lessens its utility ^35, 36^. The YEARS clinical decision rule (diagnosed DVT, hemoptysis, clinician feels PE is the most likely diagnosis) also appears to be a promising, relatively easy to implement algorithm; van der Hulle and colleagues reported a 14% decrease in computed tomographic angiography (CTA) exams required to rule out suspected PE when YEARS was paired with D-dimer level testing ^37^. Furthermore, in the ADJUST-PE study, the use of an age-adjusted D-dimer (rather than a fixed D-dimer cutoff of 500 μg/L) combined with a pre-test clinical probability assessment was associated with a larger number of patients in whom PE could be considered ruled out with a low likelihood of subsequent clinical VTE ^38^. It should be noted that recent data suggest the utility of a low clinical pre-test probability combined with D-dimer <1,000, as well as moderate clinical pre-test probability combined with D-dimer <500, in excluding PE and avoiding the need for chest imaging ^39^.

Finally, clinical gestalt is crucial and would be expected to increase with clinical experience. It has been suggested that gestalt can outperform clinical probability scoring models in suspected acute PE ^40^. Clinical probability tables are shown in Table 3.

**Table 3a.** The Wells score *.

Pulmonary embolism (PE) is most likely diagnosis	Yes = 3 points
Symptoms and signs of deep vein thrombosis (DVT) present	Yes = 3 points
Heart rate >100 beats/minute	Yes = 1.5 points
Immobilization for at least 3 days or surgery in previous 4 weeks	Yes = 1.5 points
Previous, objectively diagnosed DVT or PE	Yes = 1 point
Hemoptysis	Yes = 1 point
Malignancy with treatment within 6 months	Yes = 1 point

*In the validation cohort, a score of <4.0 (PE unlikely) combined with a negative Simpli-Red D-dimer assay (not an ELISA-based assay) accurately excluded a diagnosis of acute PE in 98% of patients. As per the first three-point item in the score, gestalt is part of the method; it is not entirely objective. Furthermore, it has been suggested that commonly, this subjective three-point “PE most likely” is what tips the score in favor of PE ^30^.

**Table 3b.** The simplified revised Geneva score *.

Variable	Score
Age ≥65 years	1
Previous deep vein thrombosis (DVT) or pulmonary embolism (PE)	1
Surgery or fracture within 1 month	1
Active malignancy	1
Hemoptysis	1
Heart rate 75 to 94 beats/minute	1
Heart rate >95 beats/minute	2
Unilateral lower limb pain	1
Pain on deep palpation of lower limb and unilateral edema	1

*The Geneva score was originally designed as a somewhat complex clinical prediction rule which required arterial blood gas analysis. It was revised and ultimately simplified. The simplified Geneva score includes the same parameters as the revised score, but the score for each parameter is uniformly 1 point, and if heart rate is >95 beats/minute an additional point is added. It is suggested that the likelihood of patients having PE with a simplified Geneva score of <2 and a normal D-dimer is 3% ^31– 33^. The simplified Geneva score was validated in the ADJUST-PE study ^33^.

**Table 3c.** The PERC score *.

Age <50 years
Pulse <100 beats/minute
Oxygen saturation >94%
Absence of unilateral leg swelling, hemoptysis, recent surgery/trauma, prior deep vein thrombosis (DVT)/pulmonary embolism (PE), and oral contraceptive use

*The PERC rule was designed to rule out acute PE in patients presenting to the emergency room without further testing. The eight variables are listed above. As a diagnostic test, low gestalt clinical suspicion for PE and PERC negative status has been shown to have a sensitivity of 97.4% (confidence interval [CI] 95.8% to 98.5%) and specificity of 21.9% (CI 21.0% to 22.9%) ^34– 36^.

**Table 3d.** The YEARS score *.

Clinical signs of deep vein thrombosis (DVT)
Hemoptysis
Pulmonary embolism (PE) most likely diagnosis
D-dimer ≥500 ng/mL or ≥1,000 ng/mL †

*In patients without YEARS items and D-dimer <1,000 ng/mL, or in patients with ≥1 YEARS items and D-dimer <500 ng/mL, PE was excluded. All others had chest computed tomographic angiography (CTA). †The primary outcome was number of venous thromboembolism (VTE) events during 3-month follow up. Of 3,465 patients, PE was excluded and CTA withheld in 1,651 patients with either no YEARS items and D-dimer level of <1,000 ng/mL or ≥1 YEARS items and D-dimer level of <500 ng/mL. VTE occurred in 0.43% of patients with PE excluded based on YEARS algorithm alone and 0.84% of patients with PE excluded based on CTA ^37^.

### Biomarkers

Biomarkers for PE are nonspecific. Elevated D-dimer levels are often paired with probability models to aid in the detection of PE and have been correlated with increased mortality ^30, 41, 42^. While the negative predictive value of D-dimer testing is very high and a normal D-dimer makes acute PE or DVT unlikely, the positive predictive value of an elevated D-dimer is low; thus, D-dimer testing is not useful for confirming acute PE ^43, 44^. Therefore, the D-dimer assay is best utilized in patients with low or moderate clinical probability, and clinical probability models have been designed and validated. In such settings, a negative test is highly sensitive for ruling out acute PE ^30^. Elevated serum troponin occurs in at least 20% of patients with acute PE, but high-sensitivity assays will likely increase this value ^45– 47^. Brain natriuretic peptide (BNP) levels are elevated in roughly 45% of PE patients, indicating RV dysfunction ^47^. Elevated D-dimer, troponin, and BNP levels have all been associated with greater mortality but are nonspecific ^45– 47^. Elevated lactate levels are clearly associated with increased mortality in acute PE patients ^25, 48^. These biomarkers are valuable in identifying suspected PE patients but are not diagnostic on their own.

### Ancillary studies

Arterial blood gas analysis may be normal, particularly in younger patients without cardiopulmonary disease ^49^. In the setting of a normal or near-normal chest radiograph and significant unexplained hypoxemia, chest CTA or ventilation–perfusion (VQ) scan should be considered to rule out PE. The electrocardiogram is nonspecific in acute PE ^50^. It may be normal or may demonstrate sinus tachycardia or an atrial arrhythmia. In particular, new-onset atrial fibrillation/flutter should raise suspicion for acute PE ^51^. The S1Q3T3 pattern is nonspecific but suggests the possibility of acute PE.

### Lung imaging

Chest radiography is generally nonspecific, but signs of pulmonary infarction appear in the form of a “Hampton’s hump” (peripheral pleural-based density) or “Westermark sign” (prominent proximal pulmonary artery with peripheral hypoperfusion ^49, 52^). ** As is the case with a normal electrocardiogram, a normal chest radiograph should increase the suspicion for acute PE in a patient without a clear explanation for symptoms such as dyspnea. CTA is a highly specific imaging technique that has become the gold standard for the diagnosis of acute PE. A high-quality CTA negative for acute PE essentially rules out the diagnosis ^53^. CTA is very useful in demonstrating other potential causes of dyspnea and chest pain. CTA may be nondiagnostic because of motion artifacts or obesity ^54– 56^. If a study is suboptimal or if there is doubt, additional lung or leg imaging should be considered ^57, 58^. CTA scans ordered for non-PE-related indications have increased, and incidental PE has become a more frequent finding ^53^. Finally, dual-energy CTA offers the opportunity to examine not only pulmonary arterial filling defects but also the actual extent of lung perfusion, which may be useful in risk stratification in proven PE; however, this technique is not yet commonly used ^59^. The radiology startup Aidoc has recently received FDA clearance for an artificial intelligence (AI) technology meant to detect and triage high-risk PE patients based on radiological images, a promising development for the rapid diagnosis of such a time-sensitive condition ^60^.

The VQ scan may be used when CTA is contraindicated due to contrast allergy, renal failure, or pregnancy ^61^. Portable VQ scans can be performed when a patient is too unstable to move and may even be useful even when the chest radiograph is abnormal ^62^. Furthermore, when a critically ill patient has a VQ scan that is nondiagnostic but with mild abnormalities, it still may be adequate to rule out PE as the cause of severe pressor-dependent hypotension. VQ with single photon emission computed tomography (SPECT) allows for three-dimensional imaging and thus better characterizes mismatched defects. The literature reports superior diagnostic value and reproducibility of SPECT relative to two-dimensional VQ; however, SPECT has not been widely accepted in clinical practice ^63, 64^.

Magnetic resonance angiography takes more time to complete than CTA, and the diagnostic yield for PE has been shown to be institution dependent ^65^. With nephrogenic fibrosing dermopathy in the setting of renal insufficiency, enthusiasm has waned. This technique is very sensitive for acute DVT. However, ultrasound is simpler, faster, and adequate in the majority of cases of suspected acute DVT.

Standard pulmonary angiography has long been considered the gold standard for the diagnosis of acute PE but nowadays is generally used only in the setting of catheter-directed acute PE therapy or, for example, when assessing a patient with chronic thromboembolic pulmonary hypertension for endarterectomy or balloon angioplasty. In acute PE, chest CTA offers the advantages of being less invasive, allowing evaluation of the lung parenchyma for other disease, and enabling assessment of RV size.

### Echocardiography

Echocardiography is useful in detecting RV dysfunction which could suggest (but not prove) the presence of PE, as well as aiding in risk stratification ^66, 67^. Echocardiography may also identify emboli in-transit in the right atrium or ventricle, which makes the diagnosis of acute PE very likely in a compatible setting, but lung imaging is still indicated whenever possible ^68^.

### Compression ultrasonography

Ultrasonography of the legs, in roughly half of cases, shows DVT in the setting of acute PE and thus serves as a powerful clue in the diagnosis of PE in compatible cases. Again, it may offer support for initiating treatment of PE when lung imaging is pending or delayed ^57, 58^.

### Pregnancy

The diagnostic approach to acute PE in pregnancy should be carefully considered. Recent data emphasize that in this high-risk setting, a diagnostic strategy based on the assessment of clinical probability, D-dimer measurement, compression ultrasound, and CTA can safely rule out PE in pregnant women. As in other settings, if PE cannot be ruled out without a CTA or VQ scan, one of these should be performed ^69^.

## Clinical guidelines

Recently published 2019 guidelines from the European Society of Cardiology/European Respiratory Society ^70^ and the American Society of Hematology ^71^ offer more details and an update on both the diagnostic and the therapeutic approaches to acute PE.

## Conclusions

Acute PE is commonly missed and can be fatal. The diagnostic approach depends on a careful and expeditious history with review of risk factors, physical examination, lab test review, and proof via imaging. Certain clinical scenarios may strongly suggest the diagnosis of PE, such as sudden-onset dyspnea with clear lungs and pleuritic chest pain. However, these scenarios are nonspecific and imaging confirmation is essential. Carefully used scoring systems may help limit the overuse of diagnostic testing and appear to be underused despite their validation in the outpatient setting. Once clinical suspicion has been raised, we have excellent tools to proceed with refuting or confirming the diagnosis. Importantly, most patients who die from acute PE die before the diagnosis is made but often before it is even suspected ^72, 73^. Anticoagulation clearly reduces mortality in acute PE; thus, the earliest possible suspicion is crucial ^74, 75^.

## Abbreviations

BNP, brain natriuretic peptide; CTA, computed tomographic angiography; DVT, deep venous thrombosis; PE, pulmonary embolism; PERC, Pulmonary Embolism Rule-out Criteria; RV, right ventricular; SPECT, single photon emission computed tomography; VQ, ventilation perfusion; VTE, venous thromboembolism.
